# The embodied meaningful place framework: integrating symbolic, personal, and internal environments in virtual reality

**DOI:** 10.3389/fpsyg.2026.1891764

**Published:** 2026-09-25

**Authors:** Silvia F. M. Pizzoli, Judith Josupeit, Lucrezia Savioni, Stefano Triberti

**Affiliations:** 1 LAHTI Laboratory for Advanced Human-Technology Interaction, Pegaso University, Milan, Italy; 2 Engineering Psychology and Applied Cognitive Research, TUD Dresden University of Technology, Dresden, Germany; 3 Department of Psychology, Vytautas Magnus University, Kaunas, Lithuania

**Keywords:** embodiment, meaningful places, virtual environments, virtual reality, wellbeing

## 1 Introduction

Virtual Reality (VR) is largely employed within health interventions (e.g., psychotherapy, pain management, rehabilitation), but most applications across literature are still based on generic, one-size-fits-all products. In contrast, personalized virtual environments (VEs) have been increasingly recognized as a powerful approach to enhance emotional engagement, sense of presence, and psychological effectiveness in VR interventions. By integrating autobiographical cues and user-relevant elements, personalized VR can facilitate emotional regulation, promote relaxation, and increase users' involvement in the experience (Pardini et al., 2022; Pizzoli et al., 2019). For example, environments constructed from personal life events or incorporating individual sensory cues can enhance emotional vividness and the sense of “being there,” which is a key mediator of VR outcomes (Baños et al., 2004; Pizzoli et al., 2022; Riva et al., 2007), while personalized environments have been associated with increased engagement, pleasure, and relaxation compared to non-personalized scenarios (Pardini et al., 2022), consistent with evidence of differential responses across relaxation modalities (Pizzoli et al., 2020). Moreover, tailored virtual stimuli can elicit intense responses: for example, VR scenarios derived from firefighters' experiences activated cognitive schemas and evoked stronger emotional reactions than generic environments (Lipp et al., 2021). However, highly personalized approaches often rely on extensive user-centered design processes, including autobiographical interviews, detailed reconstruction of personal environments, and adaptive systems, which can be resource-intensive and difficult to scale across users and clinical contexts (Maples-Keller et al., 2017; Pizzoli et al., 2019). These limitations may restrict the implementation of meaningful VR interventions.

In the present opinion piece, we will explore further the potentialities of VR, by proposing a framework that encompasses personalization but goes beyond it, to include the development of VEs based on shared symbolisms and archetypes, as well as the virtual representation of psychophysiological processes and inner experiences (“Internal State Externalization”).

## 2 Meaningful places and virtual reality: toward archetypal VR

Knaps et al. (2022) define meaningful places as locations that are either perceived as significant or carry socially constructed meanings accompanied by evaluative attachments. These meanings are not inherent to the physical environment alone but are shaped through social contexts and interpersonal relationships, regardless of being located in the real world or virtually mediated (Agnew, 2014).

Using an inductive approach, Gustafson (2001) developed a three-pole model to conceptualize meaningful places, comprising the poles of self, others, and environment. Following this perspective, meaningfulness emerges through interpretation and interaction of all three poles: places become meaningful through lived experience, the differentiation from others, and features of the environment.

On the individual level, the intrinsic need of humans to explore environments and interpret their perceptions is fundamental, for example in cognitive and perceptual dimensions like mystery, complexity, legibility, and coherence (Stamps, 2004).

In addition to the meaningfulness derived from interaction with and memories of places, physical features of the place influence its evaluation (Sebastien, 2020). Some preferences for places seem universal, for example, recreational areas or places allowing for refuge as well as those inducing feelings of presence are preferred, whereas places that might be threats to survival due to gloominess, less resources or unfamiliarity are avoided (Akcelik et al., 2024; Bennett, 2019; Johnsen, 2025; Yuan et al., 2023). Despite this universality, the evaluation is always in relation to their respective affordances. For example, different types of landscapes can be clustered into two branches associated with Jung's concept of archetypes (i.e., evolutionarily shaped patterns of experience): 1. demanding landscapes (e.g., threatening nature) and 2. restorative landscapes (e.g., recovery; (Ottosson and Grahn, 2021), which is why these results stress the importance of cross connections between Gustafson's three poles, e.g., self-environment for distinct behaviors in specific places.

These connections not only influence how a place is perceived in reference to the outside world, but also the subjective experience of being in it, or sense of presence, a psychological experience of “being there”. Jaalama et al. (2021) investigated the reciprocal relationship between the perception of a place and the sense of presence: a weak sense of presence can hinder the development of an adequate perception of a place, and vice versa.

But how is presence relevant to establish whether a place is meaningful? At the theoretical level, presence theories can be divided into “media” and “inner” theories (Coelho et al., 2006). Classical media presence theories considered sense of presence the result of interacting with a given medium, and generally defined it as an *illusion* to be in a simulated environment, mostly determined by technical sophistication of VR (Lombard and Ditton, 1997; Slater and Steed, 2000). Instead, inner presence theories consider sense of presence a fundamental function of human cognition, that locates the agent within an environment and therefore allows him or her to enact intentions and define courses of action: such primary sensation exist independently of VR but can be “tricked” by it as VR, according to Riva and colleagues (Riva et al., 2019; Triberti et al., 2025), replicates fundamental mechanisms of the human brain, namely embodied simulations, anticipating sensory information and allowing for predictions of the effects of our actions and body movements inside a space.

By recognizing the importance of the agent's contribution, inner presence theories help explaining the experimental evidence showing that sense of presence can be influenced by individual differences and personality traits (Alsina-Jurnet and Gutiérrez-Maldonado, 2010; Wallach et al., 2010), users' knowledge, habits and expectations (Ciarmoli et al., 2024), emotions (Baños et al., 2004; Wirth et al., 2012), personal meaning and significance of contents showed in a virtual simulation (Anderson et al., 2017; Cahill and Cummings, 2023), along with narratives that provide meaning to users' actions (Gorini et al., 2010; Riches et al., 2019). Sense of presence is often considered a marker of VR efficacy (Triberti et al., 2025), but feeling “there” is not sufficient for a virtual space to become a meaningful place. Meaning also depends on users' interpretation of environmental, autobiographical, symbolic, and embodied cues; these meaningful contents may, in turn, strengthen presence by making the virtual situation coherent and significant (Gorini et al., 2011; Riches et al., 2019).

## 3 Internal state externalization: the embodied virtual self

Internal State Externalization refers to the transformation of physiological, emotional, and symbolic inner processes into embodied and navigable virtual places, allowing individuals to perceive, interact with, and reconstruct aspects of their inner experience through spatial and sensorimotor engagement.

This proposal could be better understood through the lens of the Embodied Cognition (EC) paradigm. According to the seminal work by Lakoff and Johnson (2008), concepts are rooted in metaphors that draw meaning from sensorimotor experiences (e.g., strength, moving toward, containing, etc.); furthermore, as pointed out by Varela and colleagues (Varela et al., 2017; Gallagher and Lindgren, 2015), the environment (and its meaning) is not pre-given data that the organism's mind may represent more or less accurately, but rather it emerges from the structural coupling between organism and the environment itself (*enaction*). Recent developments in EC have highlighted suggestions for design: as Malafouris (2013) notices in his Material Engagement Theory, the shape of something we make (e.g., a ceramic object; a virtual environment) is not the result of a mere mental plan to be executed, but rather of the interaction between the body and the tools and materials, that continually influence cognition. Consistently, according to Dourish (2001), meaning is not *transmitted* but created through physical and social interaction. EC may suggest that a place can be “meaningful” not because it just conveys or represents rich symbolism, but in the sense that it reproduces complex, lived sensations the way our own body does. This is consistent with the prescriptions of *soma design* (Höök, 2018), an approach to interaction design that shifts focus from symbolic, language-oriented interfaces to the felt, sensory, first-person experience of the body (to the point of encouraging designers to explore their bodily experience as a source for their creative products).

Internal states such as breathing, emotional tension, or vulnerability can thus be transformed into navigable environments, for example, by representing one's breath as an expanding landscape or an emotional wound as a space. Because bodily and interoceptive signals contribute to emotion and embodied self-representation (Craig, 2002; Seth, 2013), their spatialization in VR may make internal experience perceptible and physically situated through sensorimotor interaction (Riva et al., 2007). Importantly, this externalization need not be literal: VEs may translate internal states into symbolic or metaphorical spatial forms, allowing users to interact not only with physiological signals but also with their affective and subjective meaning. Symbolic and suggestive virtual spaces have been shown to evoke strong emotional and cognitive responses and to activate personally meaningful schemas, even when represented through minimal cues (Lipp et al., 2021). This capacity fits with broader evidence that immersive VEs can elicit awe, emotional transformation, and changes in self-perception through embodied interaction with meaningful virtual spaces (Blum et al., 2019; Chirico et al., 2017).

Furthermore, the integration of physiological signals into VR enables environments and virtual bodies to dynamically respond to users' internal states, enhancing embodiment and self-awareness. For example, biofeedback-driven virtual avatars that visually reflect users' heart activity have been shown to alter body perception and emotional experience, demonstrating that physiological signals can be externalized and experienced as part of the virtual body (Aspell et al., 2013). Similarly, immersive VR biofeedback systems can dynamically integrate physiological signals to support emotional regulation and enhance embodied awareness (Blum et al., 2019).

Together, these elements support Internal State Externalization as a conceptual hypothesis: VR may enable internal states to be transformed into embodied and symbolically meaningful places, but its effects on emotional regulation, self-exploration, and therapeutic intervention require empirical testing.

In this sense, the externalization of inner processes into navigable symbolic environments can be conceptually linked to imaginative and projective techniques, as well as to therapeutic approaches that focus on bodily experience and inner states. Similar to guided imagery, imagery rescripting, and body-focused therapeutic methods, these VEs may support the exploration, modulation, and re-signification of internal experiences by making implicit sensations and emotions perceptible, manipulable, and spatially organized (Arntz, 2012; Gendlin, 1996). Internal State Externalization thus represents the embodied/internal dimension of the Embodied Meaningful Place Framework introduced below.

## 4 The embodied meaningful place framework: three sources of meaning

The Embodied Meaningful Place Framework proposes three complementary sources through which a virtual space may become psychologically meaningful: autobiographical, shared symbolic, and embodied/internal meaning. Accordingly, the three dimensions (personalization, symbolism, and embodiment/internal state externalization) are distinguished by the primary source of meaning they engage, while remaining dynamically interconnected (Figure 1).

**Figure 1:**
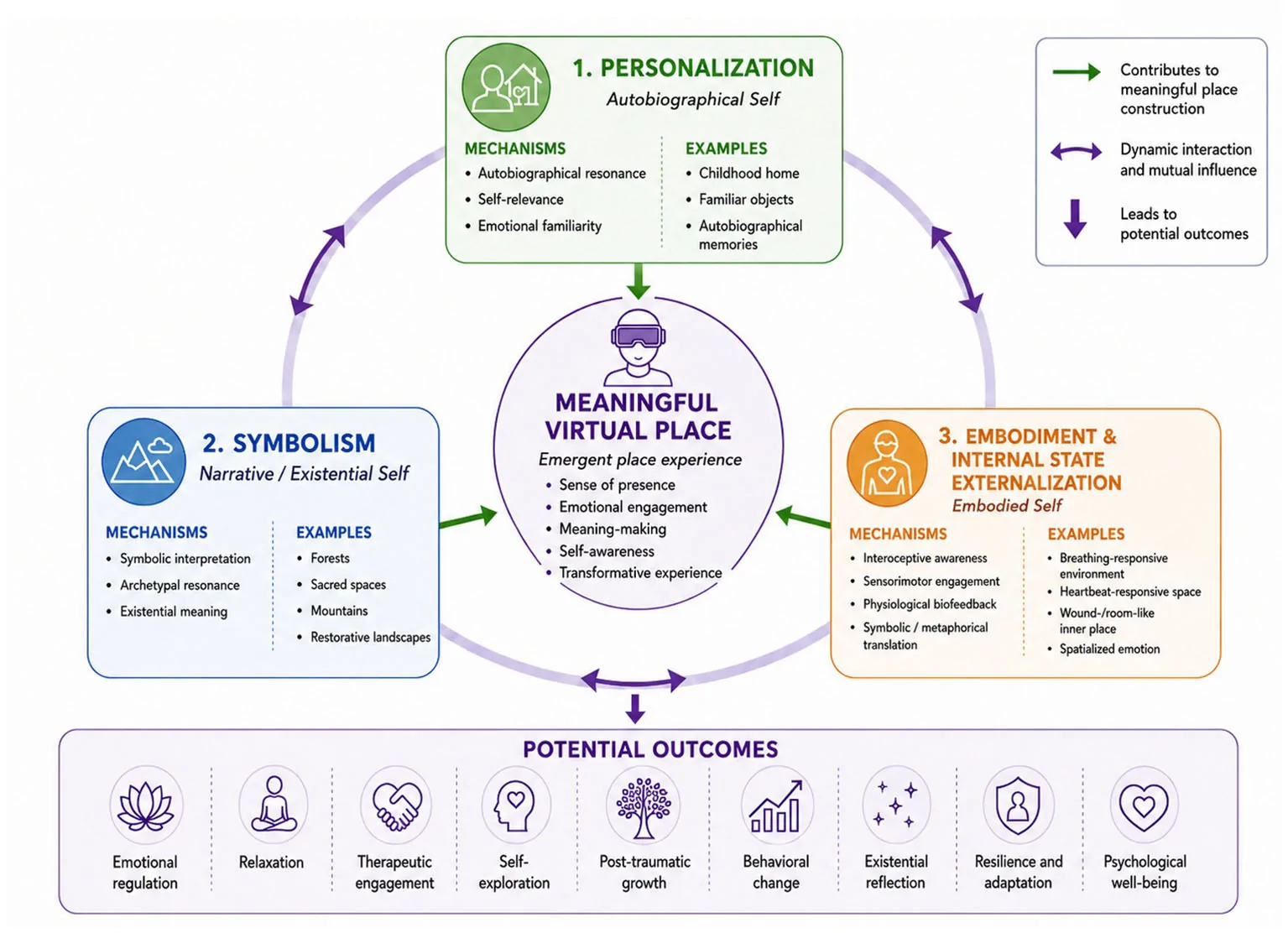
Embodied meaningful place framework. Conceptual framework describing how virtual environments may become psychologically meaningful places through the dynamic interaction of three complementary sources of meaning: personalization (*autobiographical self* ), symbolism (*narrative/existential self* ), and embodiment/internal state externalization (*embodied self* ). These dimensions draw primarily on autobiographical and identity-relevant cues, shared cultural and metaphorical meanings, and bodily, interoceptive, physiological, and affective experience, respectively. Bidirectional links indicate their dynamic interaction and mutual influence rather than independent pathways. Their integration contributes to the emergence of meaningful virtual places and may support psychological and therapeutic outcomes.

### 4.1 Personalization (personalized VR, autobiographical self)

Meaning is grounded primarily in the individual's history, identity, memories, and personally relevant cues. Although autobiographical personalization can enhance emotional engagement and relaxation, resource-intensive user-centered design may limit scalability.

### 4.2 Symbolism (archetypal VR, existential self)

Meaning is grounded primarily in shared cultural, archetypal, or metaphorical schemas that do not require reconstruction of a specific autobiographical place. Restorative natural landscapes or awe-inspiring spaces may therefore evoke emotional and self-transcendent responses through more scalable standardized environments.

### 4.3 Embodiment and internal state externalization (embodied VR, embodied self)

Meaning is grounded primarily in bodily, interoceptive, physiological, or affective states. Processes such as breathing or emotional tension can be spatialized into navigable environments and made available for sensorimotor interaction.

These dimensions can coexist within the same VE. For instance, a standardized symbolic forest may become individually meaningful by responding dynamically to the user's physiological signals, combining shared symbolism and embodied experience without extensive autobiographical reconstruction.

These propositions remain conceptual and require empirical testing. Their relevance may vary across cultures and individuals, given differences in place meaning, symbolic interpretation, autobiographical meaning-making, and interoceptive experience (Manzo, 2005; Seth, 2013). Embodied representations may also be unsuitable or distressing for some clinical populations. Finally, emotionally salient VEs raise ethical issues concerning informed consent, emotional safety, and unintended effects.

The framework thus integrates personal identity, shared meanings, and bodily experience as complementary routes to meaningful virtual places. Future research on virtual place-making should test the relative contribution of these dimensions to presence, the cross-cultural robustness of symbolic environments, whether embodiment can compensate for reduced personalization, and how meaningful virtual places should be operationalized and empirically assessed.
